# Selenite Enhances Immune Response against *Pseudomonas aeruginosa* PA14 via SKN-1 in *Caenorhabditis elegans*

**DOI:** 10.1371/journal.pone.0105810

**Published:** 2014-08-22

**Authors:** Wen-Hsuan Li, Chun-Han Chang, Chi-Wei Huang, Chia-Cheng Wei, Vivian Hsiu-Chuan Liao

**Affiliations:** Department of Bioenvironmental Systems Engineering, National Taiwan University, Taipei, Taiwan; Taipei Medical University, Taiwan

## Abstract

**Background:**

Selenium (Se) is an important nutrient that carries out many biological processes including maintaining optimal immune function. Here, inorganic selenite (Se(IV)) was evaluated for its pathogen resistance and potential-associated factors in *Caenorhabditis elegans*. The immune effects of Se(IV) were investigated by examining the responses of *C. elegans* to *Pseudomonas aerugonisa* PA14 strain.

**Principal Findings:**

Se(IV)-treated *C. elegans* showed increased survival under PA14 infection compared with untreated controls. The significant pathogen resistance of Se(IV) on *C. elegans* might not be attributed to the effects of Se(IV) on PA14 as Se(IV) showed no effect on bacterial quorum-sensing and virulence factors of PA14. This study showed that Se(IV) enhanced the expression of a gene pivotal for the innate immunity in *C. elegans*. The study found that the pathogen-resistant phenotypes contributed by Se(IV) was absent from the *skn-1* mutant worms. Moreover, Se(IV) influenced the subcellular distribution of SKN-1/Nrf in *C. elegans* upon PA14 infection. Furthermore, Se(IV) increased mRNA levels of SKN-1 target genes (*gst-4* and *gcs-1*).

**Conclusions:**

This study found evidence of Se(IV) protecting *C. elegans* against *P. aeruginosa* PA14 infection by exerting effects on the innate immunity of *C. elegans* that is likely mediated via regulation of a SKN-1-dependent signaling pathway.

## Introduction

Dietary selenium (Se), an essential trace mineral, is involved in several key metabolic activities via selenoproteins that are essential for human health to protect against oxidative damage and to maintain optimal immune function [1]. Thus, Se plays an important role in many crucial cellular processes in nearly all tissues and cell types including those involved in innate and adaptive immune responses [2], [3], [4].

Dietary Se and selenoproteins are not only important for initiating or enhancing immunity, but also involved in immuno-regulation, which is crucial for preventing excessive responses that may lead to autoimmunity or chronic inflammation [5], [6]. Se deficiency exacerbates flu symptoms and alters cytokine responses in mice fed a Se-deficient diet compared with those on an adequate Se diet [7]. Dietary Se supplementation has been shown to attenuate the lipopolysaccharide-induced expression of pro-inflammatory agents, cyclooxygenase-2 and tumor necrosis factor-alpha in macrophages [8]. Se deficiency also causes hyper-oxidant production in T cells, which results in suppression of T cell proliferation [9]. In human immunodeficiency virus (HIV)-infected children, low levels of plasma Se were significantly and independently related to mortality and faster disease progression [10]. Se deficiency is also linked to the occurrence, virulence, or disease progression of some viral infections, such as hepatitis virus (B or C) [11], [12]. These findings demonstrate an emerging and important role for Se in immune system function and this essentiality of Se has also been summarized by another report [13]. However, whether the mechanisms involved in immune systems are regulated by Se remained to be further elucidated.

The ubiquitous bacterium, *Pseudomonas aeruginosa*, is an opportunistic human pathogen commonly causing nosocomial contamination in medical care facilities [14], [15], [16]. *P. aeruginosa* may also cause serious infection in immunocompromised, HIV, and cancer patients, resulting in morbidity and mortality [16], [17]. Pathogenesis of *P. aerugonisa* is regulated by secreted virulence factors which include secondary metabolites (e.g., pyocyanin and hydrogen cyanide) and bacterial enzymes such as elastase and alkaline protease [18], [19], [20]. *P. aerugonisa* also adopts the biofilm mode of growth to make the bacteria recalcitrant to antibiotic treatments and to increase pathogenesis [21], [22]. Moreover, to facilitate the establishment of infection, *P. aerugonisa* produces both cell-associated biofilm and extracellular virulence factors regulated by two well-defined quorum-sensing systems, namely *lasIR* system and *rhlIR* system, which are cell-to-cell communication systems of the bacteria [23], [24].

*P. aeruginosa* causes lethal infection not only to human, but also to the nematode *C. elegans*, a powerful model organism for studying developmental biology and host-pathogen interactions [18], [25], [26]. *C. elegans* can be infected with numerous human pathogens and has a high degree of conservation in innate immune signaling pathways of mammals; hence, it has been widely employed to examine virulence factors, host innate defense mechanism, and drug discovery [18], [27], [28]. *P. aeruginosa* strain PA14 is a clinical isolate that can infect *C. elegans*; and screening assays of this pathosystem have been established to identify novel virulence factors and innate immune mechanisms [18], [29].

In view of the effects of Se on immune systems, it was hypothesized that pretreatment with Se(IV) can improve the survival of *P. aeruginosa* strain PA14-infected worms. Herein, the protective potential of Se(IV) on *C. elegans* under *P. aeruginosa* infection was investigated. In addition, the effects of Se(IV) on the secreted virulence factors, biofilm formation, and quorum sensing of *P. aeruginosa* were also examined Moreover, immune response gene regulation that may be responsible for the Se(IV)-induced protection of *C. elegans* against PA14 infection was explored. Finally, the possible regulatory mechanism in Se(IV)-elicited immune response in *C. elegans* was investigated.

## Materials and Methods

### *C. elegans* and bacterial strains

Inorganic selenite (Na_2_SeO_3_, Se(IV)) was purchased from Sigma-Aldrich (Poole, Dorset, UK). Strains used in this study were Bristol N2 (wild-type); EU1, *skn-1 (zu67)*; LD1, IdIs7 [*skn-1*B/C::GFP; pRF4(rol-6(su1006))] [30]; SAL105, *pha-1 (e2123)*; and denEx2[*lys-7*::GFP + pha-1(+)] [31]. All *C. elegans* strains used in this work were provided by the *Caenorhabditis* Genetics Center. *C. elegans* was normally maintained and assayed (unless otherwise stated) at 20°C on nematode growth medium (NGM) agar plates using *E. coli* OP50 bacteria as food source. *Pseudomonas aeruginosa* strain PA14 (referred hereafter as PA14) was grown with aeration at 37°C overnight in King's B broth (KB broth) [32].

### *C. elegans* slow-killing assay

For PA14 infection, the *C. elegans* slow-killing assay was adapted from the previous studies [18], [29]. Briefly, synchronized L1 larvae (wild-type or mutants) were pretreated with Se(IV) of various concentrations in liquid S-basal medium containing *E. coli* OP50 bacteria at 10^9^ cells/ml for 72 h at 20°C. Subsequently, adult *C. elegans* was subjected to the slow-killing assay using the modified nematode growth medium (0.35% peptone instead of 0.25%, NG medium) [33]. Treatments were conducted by diluting the overnight PA14 culture with NG medium to OD_600_ 0.1 as the assay medium containing Se(IV) of various concentrations. The Se(IV)-containing media were then aliquoted 500 µl/ well in a 24 well plate. About 20 worms were transferred to each well and incubated at 20°C; and the survival of the infected *C. elegans* was scored at 1-day intervals. At least three biological and three technical replicates were performed for each experiment.

### Total protease activity assay

Protease activity was examined by measuring the skimmed milk hydrolysis efficacy of the secreted protease in the bacterial culture [34]. PA14 was cultured in KB broth without or with Se (IV) (0.01 µM) overnight at 37°C. A 100-µl aliquot of PA14 KB culture with or without Se(IV) was added to 900 µl of skimmed milk (0.5% (w/v)) in Tris HCl buffer (50 mM, pH 8). Total protease activity was determined by measuring the absorbance at OD_600 nm_ at 24-h incubation. The protease activity was expressed as OD 600 per µg of protein [34]. At least three biological and three technical replicates were performed for each experiment.

### Microtiter plate biofilm formation assay

Assay of the biofilm-forming ability of the bacteria was adapted from previous studies [35], [36]. To assay biofilm formation, overnight KB broth cultures without or with Se (IV) (0.01 µM) were diluted to 1∶100 into fresh KB broth containing 0.01 µM Se(IV) or distilled water as control; and 100 µl of freshly inoculated culture was then dispensed into tissue culture-treated, 96-well polystyrene microtiter plates (Costar 3599, Corning Inc., NY, USA). The plates were incubated at 37°C for 24 h with well-controlled humidity without agitation. Biofilm formation was observed by staining with 0.1% (w/v) crystal violet at room temperature for 15 min. Subsequently, the wells were washed with distilled water to remove cells and residual dye. Crystal violet from the biofilms was eluted by absolute ethanol and then the solubilized dye was measured at OD_590 nm_. At least three biological and three technical replicates were performed for each experiment.

### Quantitative real-time RT-PCR analysis of *C. elegans* and *P. aeruginosa* PA14

For *C. elegans* preparation, wild-type *C. elegans* was treated similar to that described in previous sections except that the nematodes were collected on the first day of PA14 infection. For bacteria preparation, PA14 was cultured in KB broth without or with Se(IV) (0.01 µM) overnight at 37°C. Subsequently, the overnight PA14 cultures were centrifuged for 10 min at 1,500×*g* to collect the pellet. Total RNA from *C. elegans* or PA14 pellet was isolated using TRIzol according to manufacturer's instructions (Invitrogen, Carlsbad, CA, USA). cDNA was synthesized using Super-Script III First-strand synthesis super-Mix for qRT-PCR according to manufacturer's instructions (Invitrogen). The qRT-PCR reaction was performed on a Step One real-time cycler (Applied Biosystems, Carlsbad, CA, USA) using a SYBR Green qPCR kit (Affymetrix, Inc., Cleveland, Ohio, USA). mRNA levels were normalized to that of ACT-1 for *C. elegans* and 16S rRNA for PA14, respectively. Primer sequences used for qRT-PCR are listed in Table S1. The fold change was normalized to that observed in untreated *C. elegans* samples or untreated PA14 sample. At least three biological and three technical replicates were performed for each experiment.

### Fluorescence analysis of transgenic SAL105 (*lys-7*::GFP) *C. elegans*

Synchronized L1-stage transgenic SAL105 (*lys-7*::GFP) was cultured as described in previous sections. SAL105 cultures without or with Se(IV) (0.01 µM) were fed with OP50 or PA14, respectively, at 20°C for 24 h. The expressions of LYS-7 were directly measured by observing the reporter green fluorescent protein (GFP) fluorescence. Fluorescence in 20 nematodes randomly selected from each set of treatments was analyzed using microscopy [37]. At least three biological and three technical replicates were performed for each experiment.

### SKN-1 localization assays

Synchronized L1-stage transgenic LD1 (SKN-1::GFP) [30] was incubated in liquid S-basal containing *E. coli* OP50 bacteria at 10^9^ cells/mL without or with Se (IV) (0.01 µM) for 72 h at 20°C. Subsequently, LD1 was fed with OP50 or PA14 for 5 h [38], [39]. Subsequent to this treatment, 20 nematodes randomly selected from each set of treatments were mounted onto microscope slides coated with 2% agarose, anaesthetized with 2 mM sodium azide, and capped with coverslips. The subcellular SKN-1 distribution was analyzed by fluorescence microscopy [37]. Expression patterns of LD1 worms were classified into three categories (low, medium, and high) with respect to major localization of the SKN-1::GFP fusion protein in intestinal cells. “Low” refers to animals in which SKN-1::GFP was detected in less than 5 intestinal nuclei; “medium,” SKN-1::GFP detected in 5–15 intestinal nuclei; and “high,” SKN-1::GFP present in more than 15 intestinal nuclei [39]. At least three biological and three technical replicates were performed for each experiment.

### Data analysis

Statistical analysis of data was performed using SPSS, version 17.0 (SPSS Inc, Chicago, IL, USA). Results are expressed as the mean ± standard error of mean (SEM). The statistical significance of differences between the experimental groups was analyzed by one-way ANOVA and LSD post hoc test. Statistical differences were indicated at *p*<0.05 (*), *p*<0.01 (**) or *p*<0.001 (***) (see figures).

## Results

### Se(IV) protects wild-type *C. elegans* against *P. aeruginosa* PA14 infection

**Figure 1:**
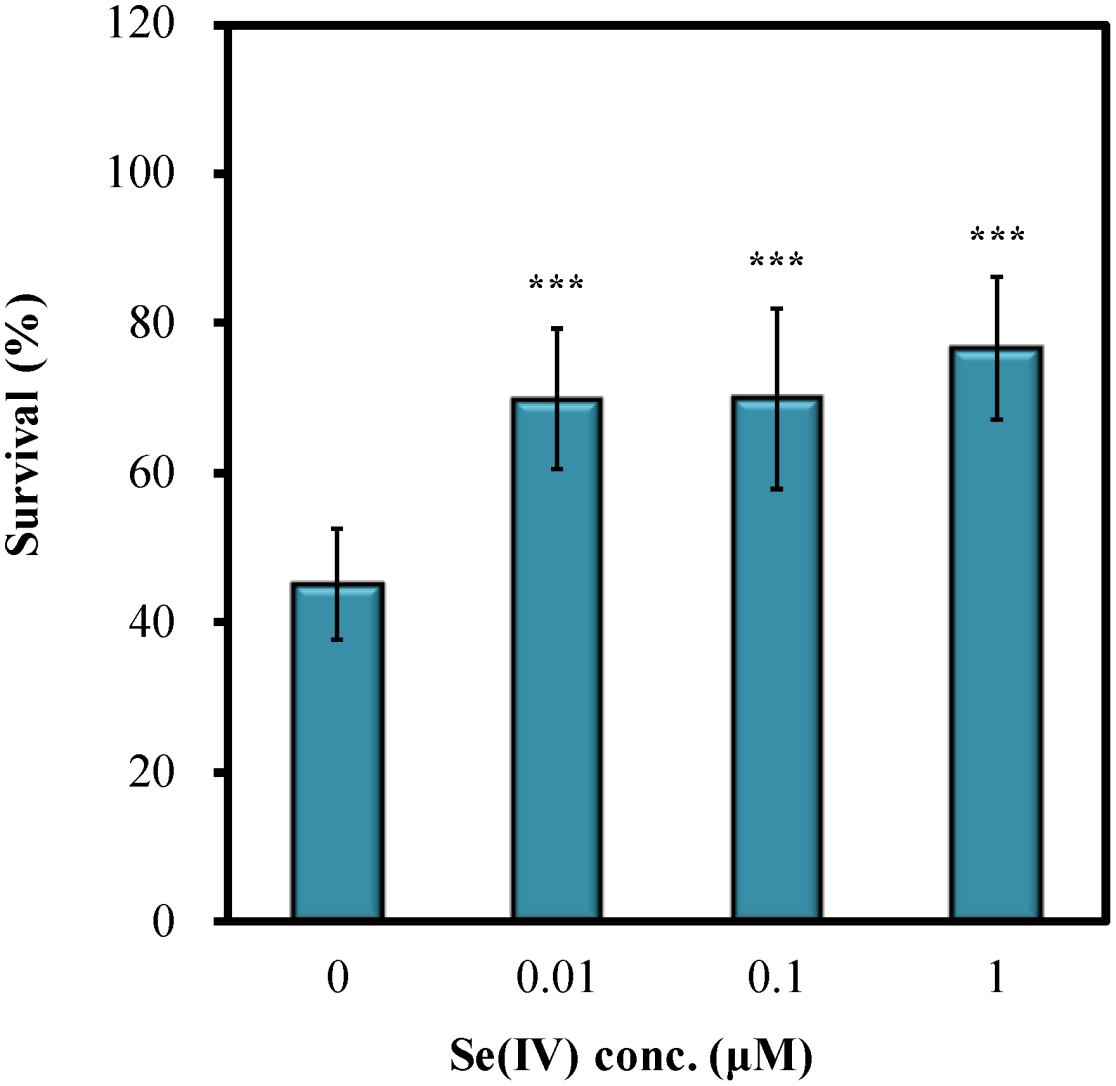
Effects of Se(IV) on survival of wild-type *C. elegans* N2 under *P. aeruginosa* PA14 infection. Synchronized wild-type L1 larvae were pretreated with Se(IV) of various concentrations (0, 0.01, 0.1, and 1 µM) for 72 h at 20°C. Subsequently, adult worms were prepared for PA14 infection. Survival of the PA14-infected worm population was scored at 1-day intervals. Graph shows the survival of *C. elegans* with or without Se(IV) treatment on the 3^rd^ day of infection as the mean ± standard error of mean (SEM). Experiments were independently performed at least three times and approximated 60 worms for each treatment were scored in each experiment. Differences compared with the control (0 µM) were considered statistically significant at *p*<0.001 (***) by one-way ANOVA and LSD post hoc test.

Previous research indicated that trace amount of Se(IV) exerts beneficial effects on development, reproduction, cholinergic signaling, neuroprotection, and oxidative stress defense in *C. elegans* [37], [40], [41]. In the present study, Se(IV)'s protective action against pathogen infection in *C. elegans* was further explored. To evaluate the effects of Se(IV), *P. aerugonisa* strain PA14 was used as the target pathogen. Wild-type N2 synchronized L1 larvae were pretreated with various concentrations of Se(IV) for 72 h at 20°C followed by PA14 infections for 72 h. Figure 1 showed that Se(IV) significantly enhanced the survival of the wild-type N2 nematodes upon PA14 infection compared with that of the control (0 µM Se(IV)). Only about 45% of PA14-infected worms survived in the control group (0 µM), whereas Se(IV)-treated nematodes showed 25% to 30% higher survival than the control group (Fig. 1). Taken together, the results indicated that Se(IV) protects wild-type *C. elegans* against *P. aeruginosa* PA14 infection.

### Se(IV) does not affect quorum-sensing and virulence factors of PA14

**Figure 2:**
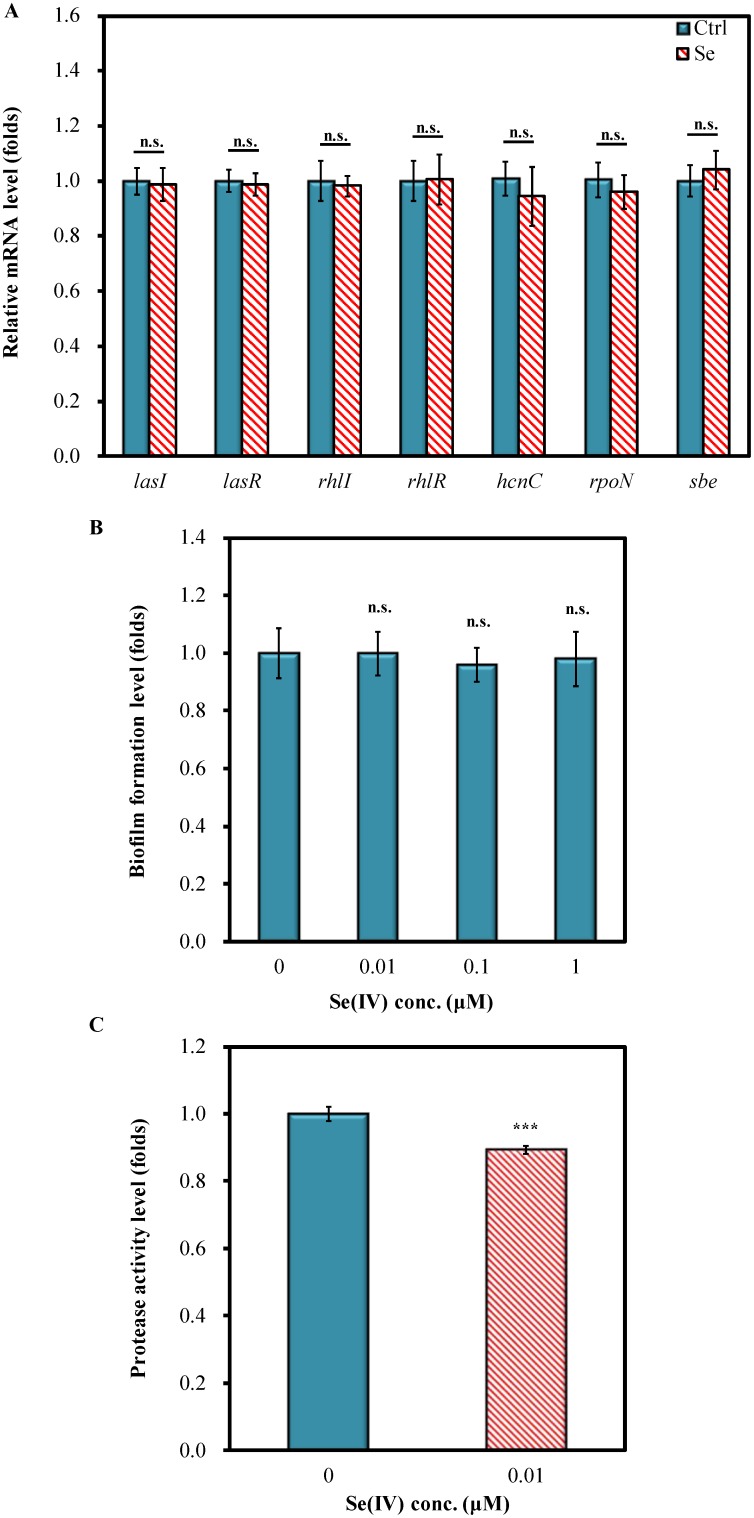
Effects of Se(IV) on quorum-sensing and virulence factors of PA14. (A). Gene expression of quorum-sensing genes and virulence factor genes of PA14 without or with Se(IV) (0.01 µM). mRNA levels were determined by quantitative real-time PCR (qRT-PCR). All measurements were normalized to mRNA levels of 16S rRNA for PA14, and fold change of each gene was normalized to that observed in control (0 µM) samples. (B). Influence of Se(IV) treatment on PA14 biofilm formation. Biofilm formation was stained with 0.1% (w/v) crystal violet and determined by measuring the absorbance at OD_590_. Fold change of each Se(IV) treatment was normalized to that observed in control (0 µM) samples. (C). Effect of Se(IV) on total protease activity of PA14. The enzyme activity was determined by measuring the absorbance at OD_600_ at 24-h incubation. Fold change of Se(IV) treatment was normalized to that observed in control (0 µM) samples. Results are presented as mean ± standard error of mean (SEM). Experiments were independently performed at least three times. Differences compared with the control (0 µM) were considered statistically significant at *p*<0.05 (*), *p*<0.001 (***) by one-way ANOVA and LSD post hoc test. n.s., no significance.

A possible mechanism that contributes to decrease the death of wild-type N2 nematodes following PA14 exposure is the inhibition of virulence factors by Se(IV) treatment. Therefore, the effects of Se(IV) on the expression of quorum-sensing genes and virulence factor genes of PA14 were examined. In all concentrations tested (0.01–1 µM), no adverse effect on PA14 growth was observed (data not shown). qRT-PCR analysis showed that 0.01 µM Se(IV) did not affect the mRNA levels of quorum-sensing genes (*lasI, lasR, rhlI*, and *rhlR*) (Fig. 2A). Moreover, the mRNA levels of several virulence factor genes, including *hcnC*, *rpoN*, and *sbe*, showed no significant changes under treatment with 0.01 µM Se(IV) in the culture medium (Fig. 2A). These results suggested that Se(IV) might not affect the pathogenesis-related genes of *P. aeruginosa* PA14.

In addition, biofilm formation and secretory virulence factors such as protease in the PA14 culture grown in King's B broth supplemented with Se(IV) were examined. The effect of Se(IV) on biofilm formation was studied by crystal violet staining. The results showed that Se(IV) did not inhibit the biofilm formation of PA14 (Fig. 2B). Moreover, the protease activity of PA14 was determined by analyzing the ability of culture supernatants to lyse skimmed milk powder via measurement of absorbance at OD_600 nm_. Se(IV) treatment slightly decreased the total protease activity of pathogen-secreted enzyme by about 10% (Fig. 2C). Taken together, the results showed that Se(IV) does not protect wild-type *C. elegans* against *P. aeruginosa* PA14 pathogenicity by inhibiting virulence factors and reducing quorum-sensing signals of PA14.

### Se(IV) enhances immune response gene expression in *C. elegans* under PA14 infection

**Figure 3:**
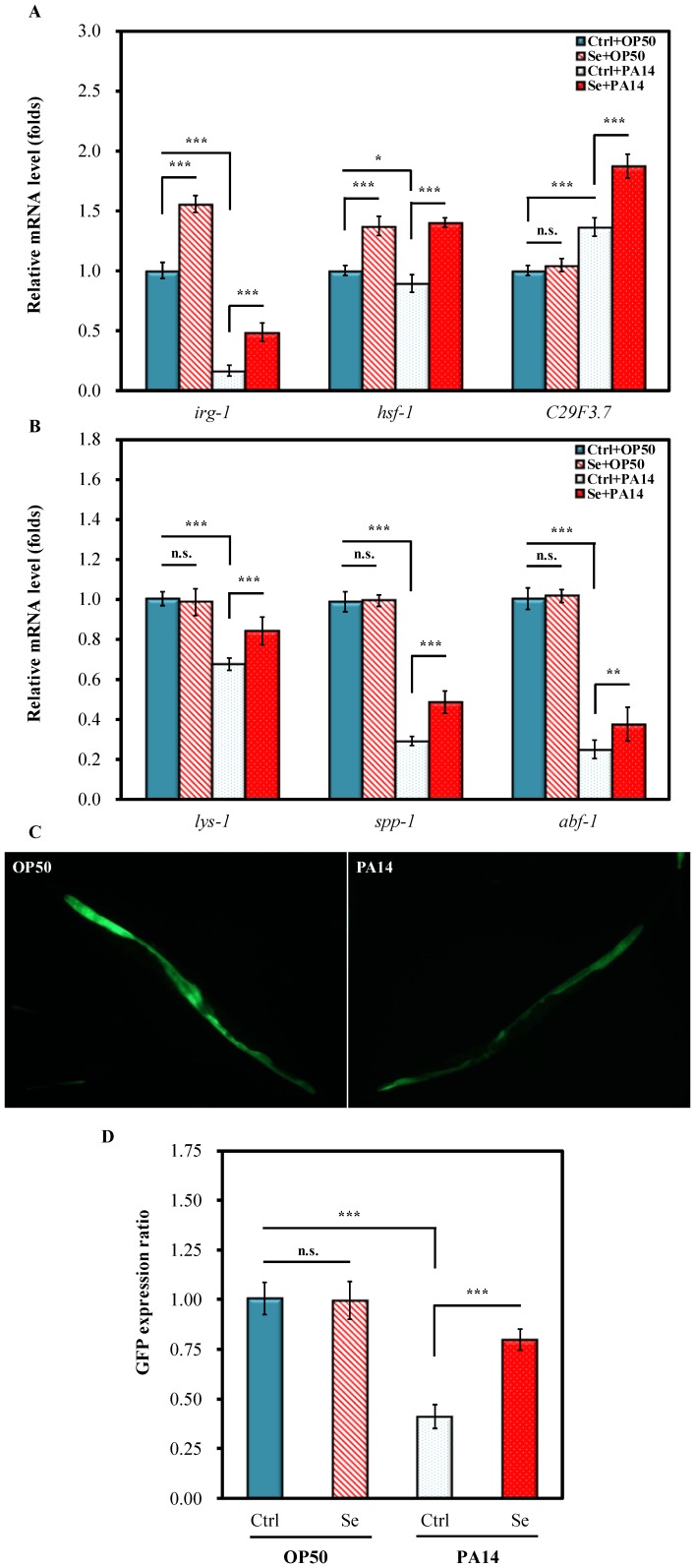
Effects of Se(IV) on immune response genes in *C. elegans*. Synchronized L1 wild-type (N2) or transgenic strain (SAL105) larvae were pretreated without (0 µM) or with Se(IV) (0.01 µM) for 72 h at 20°C. Subsequently, Se(IV)-pretreated and control (0 µM) adult worms were divided into two aliquots and fed with OP50 or PA14 for 24 h at 20°C. The relative gene expressions were then determined by quantitative real-time PCR (qRT-PCR) or by GFP quantification. (A). Relative mRNA levels of the early immune response genes in wild-type *C. elegans*. (B). Relative mRNA levels of the late immune response genes in wild-type *C. elegans*. Ctrl + OP50: worm raised with OP50 for 72 h and then exposed to OP50 for another 24 h without Se(IV) exposure; Se + OP50: worm raised with OP50 for 72 h and then exposed to OP50 for another 24 h with 0.01 µM Se(IV) exposure; Ctrl + PA14: worm raised with OP50 for 72 h and then infected with PA14 for another 24 h without Se(IV) exposure; Se + PA14: worm raised with OP50 for 72 h and then infected with PA14 for another 24 h with 0.01 µM Se(IV) exposure. All measurements were normalized to mRNA levels of ACT-1 for *C. elegans*, and fold change of each gene was normalized to that observed in “Ctrl + OP50” samples. (C). (*Left*) Representative image of transgenic SLA105 (*lys-7*::GFP) cultured in normal conditions with OP50 as food source; (*Right*) Representative image of transgenic SLA105 (*lys-7*::GFP) infected with PA14 for 24 h without Se(IV) treatment. Worms infected with PA14 for 24 h and treated with 0.01 µM Se(IV) showed increased GFP fluorescence intensity similar to that presented in (*left*). (D) Quantifications of GFP expression of *lys-7* gene in each treatment condition normalized to that of the control (Ctrl fed with OP50). Results are presented as mean ± standard error of mean (SEM). Experiments were independently performed at least three times and approximated 40 worms for each treatment were scored in each experiment. Differences compared with the control (0 µM) were considered statistically significant at *p*<0.05 (*), *p*<0.01 (**), and *p*<0.001 (***) by one-way ANOVA and LSD post hoc test. n.s., no significance.

Whether immune response gene regulation may be responsible for the Se(IV)-induced protection of *C. elegans* against PA14 pathogenicity was examined. Three early response genes: *irg-1* (infection response gene 1), *hsf-1* (heat shock factor 1), and *C29F3.7* (CUB-like domain) [42], [43] and three late response genes: *lys-1* (lysozyme-like protein), *spp-1* (saponin-like protein), and *abf-1* (antibacterial protein) [44], [45] were selected. The mRNA levels in PA14-infected and uninfected worms with Se(IV) treatment were investigated. *E. coli* OP50, the standard laboratory food for *C. elegans*, served as the uninfected control to investigate the effects of Se(IV) under normal diet. Under normal diet, the mRNA levels of all tested immune-related genes, except *irg-1* and *hsf-1*, were not significantly altered by Se(IV) compared with those of the control fed with OP50 (Ctrl + OP50) (Fig. 3A and 3B). In addition, after 24-h PA14 infection, the mRNA levels of all tested immune genes, except *C29F3.7* (Ctrl + PA14), were significantly suppressed by 20% to 80% compared with that of the uninfected group (Ctrl + OP50) (Fig. 3A and 3B). Noticeably, Se(IV) treatment led to more significant activation of all six immune genes in PA14-infected worms (Se + PA14) than in the uninfected control (Ctrl + PA14) (Fig. 3A and 3B). Taken together, the results showed that Se(IV) enhanced immunity in *C. elegans*.

To further provide evidences for the beneficial effects of Se(IV) on immune systems, transgenic strain SAL105 (*lys-7*::GFP) was employed to observe the effect of PA14 infection and Se(IV) on *lys-7* expression. It has been reported that PA14 suppresses *C elegans* immunity by repressing the expression of the lysozyme-like LYS-7 [45]. The effect of *Swietenia macrophylla* on the expression of *lys-7* was previously described [46]. The current results showed that PA14 infection diminished the overall GFP fluorescence intensity in *C. elegans*, indicating decreased expression of *lys-7* compared with that in the uninfected *C. elegans* (Fig. 3C). Furthermore, Se(IV) treatment enhanced the GFP fluorescence intensity to a level comparable to that in uninfected *C. elegans*.

The GFP fluorescence intensity in each treatment group was further quantified. Quantitated data showed that *lys-7* expression was not affected by Se(IV) under normal diet (*E. coli* OP50) but Se(IV) prevented the decrease of *lys-7* gene expression during PA14 infection (Fig. 3D). This evidence was in agreement with above qRT-PCR data. Overall, the results suggested that Se(IV) enhanced immunity in *C. elegans* via activation of the immune genes under PA14 infection.

### SKN-1 is essential for Se(IV)-induced protection of *C. elegans* against PA14 infection

**Figure 4:**
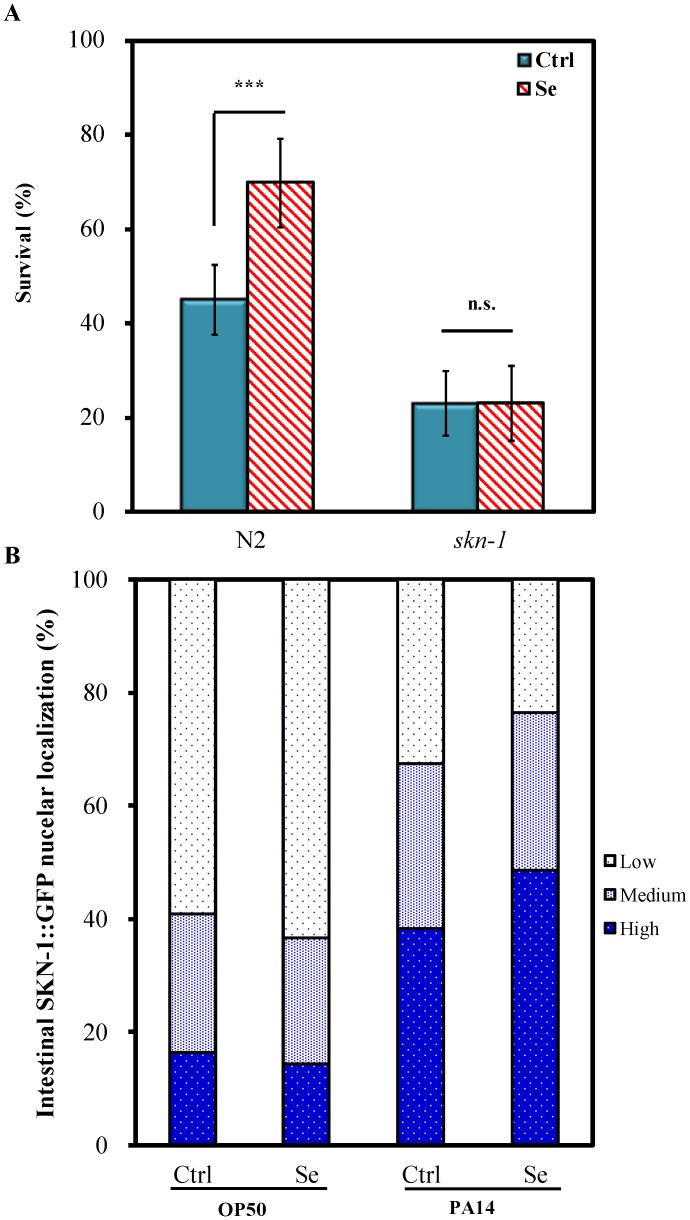
Influences of Se(IV) on *skn-1* mutant and subcellular SKN-1 localization under PA14 infection. Synchronized wild-type N2, *skn-1* mutant, or transgenic LD1 strain (SKN-1::GFP) L1 larvae were pretreated without (0 µM) or with Se(IV) (0.01 µM) for 72 h at 20°C. Subsequently, adult worms were prepared for PA14 infection. (A). Survival of the PA14-infected worm population was scored at 1-day intervals. Graph shows the survival of *C. elegans* without or with Se(IV) treatment on the 3^rd^ day of infection as mean ± standard error of mean (SEM). Experiments were independently performed at least three times and approximated 60 worms for each treatment were scored in each experiment. Differences compared with the control (0 µM) were considered statistically significant at *p*<0.001 (***) by one-way ANOVA and LSD post hoc test. n.s., no significance. (B). Expression patterns of LD1 worms were classified into three categories (low, medium, and high) with respect to major localization of the SKN-1::GFP fusion protein in intestinal cells. “Low” refers to animals in which SKN-1::GFP was detected in less than 5 intestinal nuclei; “medium,” SKN-1::GFP detected in 5–15 intestinal nuclei; “high,” SKN-1::GFP present in more than 15 intestinal nuclei [39]. Experiments were independently performed at least three times and approximated 40 worms for each treatment were scored in each experiment.

In *C. elegans*, SKN-1/ Nrf transcription factor plays an important role not only in oxidative and xenobiotic stress responses [47], [48] but also in innate immunity [38], [39]. To investigate whether Se(IV)-enhanced *C. elegans* against *P. aeruginosa* PA14 pathogenicity was modulated by SKN-1, *skn-1 (zu67)* mutant in response to Se(IV) was examined. Unlike the wild-type N2 *C. elegans* (Fig. 1), *skn-1* mutant did not show significantly increased survival after 0.01 µM Se(IV) treatment for 3 days at 20°C followed by PA14 infection compared with no treatment (Fig. 4A), suggesting that Se(IV) may provide PA14 pathogen resistance in *C. elegans* via SKN-1.

SKN-1 is a transcription factor playing multiple essential roles; and localization of SKN-1 in nuclei is an essential prerequisite for activating transcription of target genes, such as *gst-4* and *gcs-1* [30], [47], [48], [49]. Therefore, to further explore the role of SKN-1 in regulating Se(IV)-enhanced PA14 pathogen resistance, the nuclear translocation of SKN-1 was examined using the transgenic strain LD1 (SKN-1B/C::GFP). Transgenic LD1 strain was cultured to adulthood without or with Se (IV) (0.01 µM) as described above. The adult worms were then fed with *E. coli* OP50 for 5 h as uninfected controls or infected with PA14, and the patterns of SKN-1 nuclear localization in intestinal cells were scored using the fluorescence microscope. The results showed no significant difference in SKN-1 nuclear localization between untreated worms and Se(IV)-treated worms under OP50 diet (Fig. 4B). Without Se(IV) treatment, a massive accumulation of SKN-1::GFP could be observed in intestinal nuclei of PA14-infected worms compared with those fed with nonpathogenic OP50 (Fig. 4B), indicating that PA14 triggered SKN-1 nuclear localization in intestinal cells of *C. elegans*. Moreover, the results also showed increase in SKN-1 nuclear localization in intestinal cells of the Se(IV)-treated group compared with the untreated group under PA14 infection (Fig. 4B). Taken together, the results showed that the PA14 pathogen resistance in Se(IV)-treated *C. elegans* could be attributed to Se(IV)-triggered SKN-1 nuclei translocation in intestinal cells.

### Se(IV) enhances expressions of glutathione-S-transferase (GST-4) and gamma-glutamine cysteine synthetase (GCS-1) in *C. elegans* under PA14 infection

**Figure 5:**
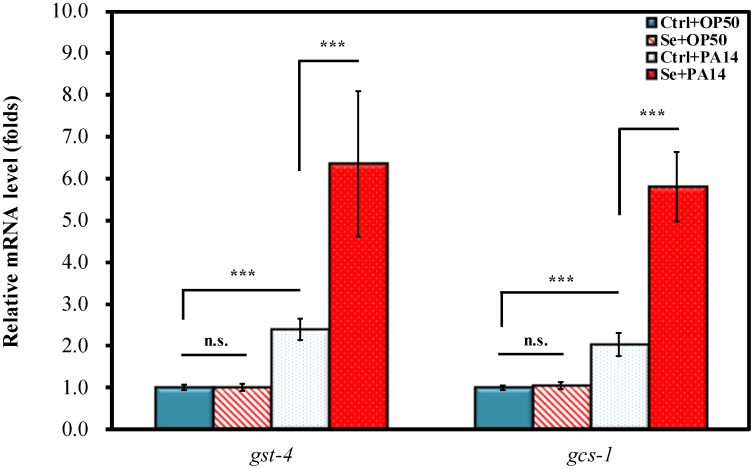
Effects of Se(IV) on expression of SKN-1 target genes upon PA14 infection in *C. elegans*. Synchronized L1 wild-type larvae were pretreated without (0 µM) or with Se(IV) (0.01 µM) for 72 h at 20°C. Subsequently, Se(IV)-pretreated and control adult worms were divided into two aliquots and fed with OP50 or PA14 for 24 h at 20°C. Subsequently, the mRNA levels of GST-4 and GCS-1 were determined by qRT-PCR. All measurements were normalized to mRNA levels of ACT-1 for *C. elegans*, and fold change of each gene was normalized to that observed in “Ctrl + OP50” samples. Results are presented as mean ± standard error of mean (SEM). Experiments were independently performed at least three times. Differences compared with “Ctrl + OP50” were considered statistically significant at *p*<0.001 (***) by one-way ANOVA and LSD post hoc test. n.s., no significance.

To elucidate whether the increase in PA14 pathogen resistance described above was due to Se(IV) regulating SKN-1-dependent gene expressions, the mRNA levels of glutathione-S-transferase (GST-4) and gamma-glutamine cysteine synthetase (GCS-1) in response to Se(IV) treatment and PA14 infection were examined. Under normal *E. coli* OP50 diet, Se(IV) did not significantly affect the mRNA levels of both *gst-4* and *gcs-1* (Ctrl + OP50 vs. Se + OP50) (Fig. 5). Upon PA14 infection, the mRNA levels of *gst-4* and *gcs-1* were significantly elevated compared with those in uninfected *C. elegans* on OP50 diet (Ctrl + OP50 vs. Ctrl + PA14, *p*<0.001) (Fig. 5). Moreover, the results showed that Se(IV) treatment caused up-regulation of *gst-4* and *gcs-1* gene expression under PA14 infection (Ctrl + PA14 vs. Se + PA14, *p*<0.001) (Fig. 5). Taken together, the results indicate that Se(IV) triggered increased expression of SKN-1 downstream target genes such as *gst-4* and *gcs-1* under PA14 pathogen infection.

## Discussion

Previous studies reported that trace amount of Se(IV) exerts multiple beneficial effects on development, reproduction, cholinergic signaling, neuroprotection, and oxidative stress resistance in *C. elegans* [37], [40], [41]. In the present study, the protective potential of Se(IV) on *C. elegans* against *P. aeruginosa* infection was further investigated. The *C. elegans*/*P. aeruginosa* slow-killing infection model was employed to test the ability of Se(IV) to induce immune responses in *C. elegans*. In addition, antibacterial potential of Se(IV) against the pathogenic *P. aeruginosa* strain PA14 was examined. The results showed that trace amount of Se(IV) protects wild-type N2 *C. elegans* against *P. aeruginosa* PA14 infection (Fig. 1), suggesting that supplementation of Se(IV) may enhance immune responses to *C. elegans*. Dietary Se has been indicated as an essential micronutrient for optimal immune responses; however, the mechanisms accounting for such requirement are not fully understood [50], [51]. A number of studies have investigated the effects of Se-deficiency and Se-supplementation on immune responses, demonstrating an elevation of both cell-mediated and humoral immune responses by increasing Se intake levels [6], [51]. Antiviral immune responses have been generally shown to be affected by host Se status. However, the effect of Se on the immune responses to non-viral pathogens is more complicated [13], [51]. Currently available data suggested that host resistance of Se to pathogen infections varies with the microorganism involved [13], [51]. Thus, Fig. 1 provided further evidence showing that supplementation of Se(IV) enhances immune responses against *P. aeruginosa* PA14 infection, suggesting the importance of Se intake to immune system of organisms.

The increased survival of PA14-infected *C. elegans* under Se(IV) treatment (Fig. 1) may result from enhanced host immune defense systems or weakened pathogen activities and viability. Recent studies from water extract of TWE made from the brown seaweed (*Ascophyllum nodosum*) and water extract of red seaweed *Chondrus crispus* (CCWE) have been shown to enhance host *C. elegans* immunity by suppressing the mRNA levels of quorum sensing and the virulence factors of PA14 [44], [52]. The antioxidant curcumin was shown to enhance *C. elegans* survival by limiting *P. aeruginosa* pathogenicity through the repression of several quorum-sensing genes [53]. Moreover, anti-quorum-sensing activity provided by extractions from plants or marine sponge-associated bacteria (e.g., *Syzygium aromaticum*, *Terminalia chebula Retz.*, and *Haliclona spp.*) was shown to improve protections of *C. elegans* against pathogen infections [54], [55], [56]. The effects of Se(IV) on both *C. elegans* and PA14 were examined in the present study. Se(IV) did not affect the growth of PA14 (data not shown). qRT-PCR analysis showed that 0.01 µM Se(IV) did not affect the mRNA levels of the tested quorum-sensing genes (*lasI, lasR, rhlI*, and *rhlR*) and virulence factor genes (*hcnC*, *rpoN*, and *sbe*) of PA14 (Fig. 2A). Moreover, PA14 grown with Se(IV) (0.01 µM) supplementation showed that Se(IV) did not inhibit the biofilm formation of PA14 (Fig. 2B), whereas total protease activity was slightly decreased by Se(IV) treatment (Fig. 2C). Therefore, Se(IV) did not protect wild-type *C. elegans* against *P. aeruginosa* PA14 pathogenicity by inhibiting virulence factors and reducing quorum-sensing signals of PA14.

Whether the increased survival of PA14-infected *C. elegans* by Se(IV) treatment (Fig. 1) was attributable to the enhancement of immune systems of *C. elegans* was examined. Repression of host defense genes is often associated with suppression of host defense pathways by the pathogen. *P. aeruginosa* infection is responsible for the impairment of host defense through the down-regulation of host's antimicrobial factors [45], [57]. The present findings showed that after PA14 infection, the mRNA levels of selected immune-related genes (namely, *irg-1*, *hsf-1*, *lys-1*, *spp-1*, and *abf-1*) (Ctrl + PA14) were significantly suppressed 20% to 80% compared with that of the uninfected group (Ctrl + OP50) (Fig. 3A and 3B) without Se(IV) treatment. The PA14-suppressed mRNA levels were attenuated when PA14-infected *C. elegans* were treated with 0.01 µM Se(IV) (Fig. 3A and 3B), suggesting that Se(IV) enhanced immunity in *C. elegans* by enhancing the immune responses of *C. elegans*. These similar effects were observed in water extracts from the brown seaweed (*Ascophyllum nodosum*) and red seaweed *Chondrus crispus* [44], [52]. PA14 infection was further shown to suppress the overall GFP fluorescence intensity in transgenic *C. elegans* carrying *lys-7*::GFP compared with that of uninfected *C. elegans*, whereas Se(IV) treatment enhanced the GFP fluorescence intensity to a level comparable to that of uninfected *C. elegans* (Fig. 3C and 3D). This similar effect was observed in *Swietenia macrophylla*, in which the plant extract restored the initially repressed *lys-7* expression in PA14-infected *C. elegans* [46], [52]. Taken together, the results showed that Se(IV) might enhance immunity in *C. elegans* via activation of immune genes under PA14 infection.

In *C. elegans*, several conserved signal transduction pathways including the mitogen-activated protein kinase (MAPK) pathways, insulin/IGF-like signaling (IIS), and TGF-β pathways are involved in the immune responses [58],[59]. Both p38 MAPK and IIS pathways regulate SKN-1 that masters both oxidative and xenobiotic stress responses in *C. elegans* [47], [48], though the role of SKN-1 in the regulation of pathogen response is not well understood. SKN-1 has recently been shown as a prerequisite for *C. elegans* pathogen resistance, suggesting SKN-1 as a regulator of the innate immunity [39]. Therefore, to gain a mechanistic view of Se(IV) regulating immune responses in *C. elegans*, whether Se(IV) is linked to SKN-1 activity in immune responses was examined. Figure 4A showed that lack of SKN-1 resulted in sensitivity to PA14 infection, a finding consistent with previous studies [38], [39]. In contrast to the observation in wild-type *C. elegans*, the enhanced survival against PA14 infection by Se(IV) was not observed in *skn-1* deletion mutant (Fig. 4A), suggesting the essential role of SKN-1 in Se(IV)-enhanced PA14 pathogen resistance in *C. elegans*.

To further validate that SKN-1 is necessary for the pathogen resistance induced by Se(IV), the effect of Se(IV) on accumulation of SKN-1 in intestinal nuclei was examined (Fig. 4B). Exposure to *P. aeruginosa* leads to SKN-1 accumulation in intestinal nuclei; and transcriptional activation of SKN-1 target genes, *gcs-1* and *gst-4*, has been described [38], [39]. Without Se(IV) treatment, SKN-1 accumulation in intestinal nuclei could be observed in PA14-infected worms compared with those on nonpathogenic OP50 diet (Fig. 4B); and Se(IV) led to further increase in SKN-1 nuclear localization in intestinal cells (Fig. 4B). These observations suggested that PA14 pathogen resistance induced by Se(IV) was due to triggered SKN-1 nuclei translocation in intestinal cells of *C. elegans*. Further evidence showed that Se(IV) significantly up-regulated the SKN-1 target genes: *gst-4* and *gcs-1* gene expression under PA14 infection (Fig. 5), suggesting that Se(IV) triggered increased expression of SKN-1 downstream target genes such as *gst-4* and *gcs-1* under PA14 pathogen infection. Taken together, the results showed that Se(IV) protects *C. elegans* from *P. aeruginosa* infection via SKN-1.

There has been increasing evidence suggesting that selenoproteins play important roles in regulating inflammation and immunity, providing important insight into mechanisms by which Se influences inflammation and immunity [13]. In contrast to results observed in other animals, the only selenoprotein in *C. elegans* TRXR-1, an ortholog of the human thioredoxin reductase-1, was shown not to directly protect *C. elegans* from oxidative stress [60], [61]. This implies a unique role of *C. elegans* selenoprotein. A recent study indicated that TRXR-1 is involved in Se(IV) regulating oxidative stress resistance in *C. elegans* [37]. The role of TRXR-1 in Se(IV)-mediated immune responses in *C. elegans* requires further elucidation.

In conclusion, the present study using the model animal *C. elegans* obtained evidence supporting a beneficial effect of Se(IV) in host immune regulation. The findings revealed Se(IV) protecting *C. elegans* against *P. aeruginosa* PA14 infection by exerting effects on the innate immunity of *C. elegans* but having no direct effects on bacterial quorum-sensing and virulence factors. Se(IV) was also found to enhance the expression of a gene pivotal for the innate immunity in *C. elegans*. Finally, mechanistic study indicated that the protective effects of Se(IV) is likely mediated via regulation of a SKN-1/Nrf-dependent signaling pathway by inducing the expression of the target genes (*gst-4* and *gcs-1*), thereby enhancing immune resistance on *C. elegans* against *P. aeruginosa* PA14 infection. These findings advance the understanding of the regulatory mechanism of Se in immune systems of intact organisms.

## Supporting Information

Table S1 **Sequences of primers used for real time PCR.**
(DOC)
