# Supplementary material for: Selenite Enhances Immune Response against Pseudomonas aeruginosa PA14 via SKN-1 in Caenorhabditis elegans
Source: PLoS One. 2014 Aug 22;9(8):e105810. doi: 10.1371/journal.pone.0105810 (PMC4141825; doi:10.1371/journal.pone.0105810)
Supplement: Table S1 — Sequences of primers used for real time PCR. (DOC) [file pone.0105810.s001.doc]

| ***C. elegans*** | **Gene** | | **Primer sequences (5’-3’)** |
| --- | --- | --- | --- |
|  | *irg-1* | forward | AAGCAGCATGCGTATTTTCA |
|  |  | reverse | GCAGCTTCTCCTTTTTCTCC |
|  | *hsf-1* | forward | CAGCCAACAGGGAATCAAAT |
|  |  | reverse | TGCTGCTCCAGAAACTGAAA |
|  | *C29F3.7* | forward | GATCGGCAACTTTACCTCCA |
|  |  | reverse | AATTGTGGCGGATATTCTGG |
|  | *lys-1* | forward | TTCGGATCTTTCAAGAAGGC |
|  |  | reverse | GGGATTCCAACAACGTAAA |
|  | *spp-1* | forward | TGAACATCGGAACTCTTTGC |
|  |  | reverse | TCAGCTCTTCCTCACACTCG |
|  | *abf-1* | forward | TGCCTTCTCCTTGTTCTCCT |
|  |  | reverse | ATCCTCTGCATTACCGGAAC |
|  | *gst-4* | forward | ATGCTCGTGCTCTTGCTGAG |
|  |  | reverse | GACTGACCGAATTGTTCTCCAT |
|  | *gcs-1* | forward | GTCGATGAAGCCAGATGGTTGT |
|  |  | reverse | CGATCGTCGACACTTGCACTAA |
|  | *act-1* | forward | GCTGGACGTGATCTTACTGATTACC |
|  |  | reverse | GTAGCAGAG CTTCTCCTTGATGTC |
| ***P. aeruginosa*** | **Gene** | | **Primer sequences (5’-3’)** |
|  | *lasI* | forward | GCTCCTTGAACACTTGAGCA |
|  |  | reverse | GCGCGAAGAGTTCGATAAAA |
|  | *lasR* | forward | CCGCCGAATATTTCCCATA |
|  |  | reverse | GATATCGGTTATCTGCAACTGCT |
|  | *rhlI* | forward | GGAGCGCTATTTCGTTCG |
|  |  | reverse | GTCTCGCCCTTGACCTTCT |
|  | *rhlR* | forward | TGCGTTGCATGATCGAGT |
|  |  | reverse | CGGGTTGGACATCAGCAT |
|  | *hcnC* | forward | GCCTGGACAGTTGGTAGGC |
|  |  | reverse | GAACAGAACCTATGACATCGTGA |
|  | *rpoN* | forward | ATACCTTCATGCGCAACCA |
|  |  | reverse | GGCTCTGCAGGCTCTTGAT |
|  | *sbe* | forward | CTCGTTGGTCTCCTCGAGTT |
|  |  | reverse | CCATCTACCAGCGTGAAGG |
|  | *16S rRNA* | forward | GATTAACGCTTGCACCCTTC |
|  |  | reverse | TAAGCACCGGCTAACTTCGT |
